# Development and Prospective Validation of Wearable Sensor-Based Gait Metric for Individuals with Lower-Limb Amputation

**DOI:** 10.3390/s26144613

**Published:** 2026-07-21

**Authors:** Christopher Bennett, Ignacio Gaunaurd, Allison Symsack, E. Brooks Applegate, Josué de León Santana, Paul Pasquina, Robert Gailey

**Affiliations:** 1 Department of Music Engineering, Frost School of Music, University of Miami, Coral Gables, FL 33146, USA; 2 Department of Physical Therapy, Miller School of Medicine, University of Miami, Miami, FL 33136, USA; 3 Research Department, Miami Veterans Affairs Healthcare System, Miami, FL 33136, USA; 4 Department of Physical Medicine & Rehabilitation, Uniformed Services University of the Health Sciences, Bethesda, MD 20814, USA; 5 Department of Education Leadership, Research, and Technology, Western Michigan University, Kalamazoo, WI 49008, USA

**Keywords:** wearable sensors, lower-limb amputation, gait analysis, inertial measurement units, machine learning

## Abstract

**Highlights:**

**What are the main findings?**

- A portable IMU-based classifier accurately identified clinically observed gait deviations in individuals with lower-limb amputation.
- A derived Gait Goodness Score differentiated functional mobility levels and was associated with established clinical performance measures.

**What are the implications of the main findings?**

- Relative outputs from machine learning classifiers can be transformed into a continuous, interpretable score of gait quality suitable for clinical monitoring.
- Wearable sensor-based gait assessment can complement standard rehabilitation outcome measures and support objective evaluation.

**Abstract:**

Lower-limb amputation is associated with persistent gait asymmetries and functional limitations that are not fully captured by conventional clinical outcome measures. This study aimed to develop and prospectively validate a wearable sensor-based Gait Goodness Score (GGS) derived from ensemble classifiers to summarize overall gait quality during supervised clinical walking. The algorithm was previously trained using inertial measurement unit data and clinically meaningful temporal–spatial features. In the present prospective, observational validation study, medically stable adults with lower-limb amputation performed supervised 10 m walk tests in outpatient rehabilitation settings, during which step-based GGS values were collected. Associations between GGS and established clinical measures, including walking velocity, Amputee Mobility Predictor (AMP) score, and Timed Up and Go (TUG) durations, were examined. GGS demonstrated significant differences across functional levels and amputation levels and showed strong associations with walking velocity and AMP score, with a significant moderate-to-fair association also observed for TUG and PLUS-M. These findings support the validity of GGS as a quantitative, sensor-derived metric of gait quality during supervised clinical walking in individuals with lower-limb amputation.

## 1. Introduction

Approximately two million individuals in the United States live with limb loss, a figure that increases by 185,000 amputations annually due to vascular disease and trauma [1,2,3,4]. Lower-limb amputation (LLA) often results in persistent gait asymmetries and compensatory strategies that evolve over time, contributing to secondary musculoskeletal injury, reduced mobility, or diminished quality of life [5,6,7,8,9,10]. Clinical gait assessment is central to prosthetic rehabilitation; however, gold-standard laboratory-based motion analysis is cost-prohibitive and time-intensive. Consequently, clinicians rely on standardized functional tests, such as the 10 m walk test (10mWT) or Amputee Mobility Predictor (AMP), and observational gait assessment to evaluate functional performance, identify gait deviations, and guide clinical decision-making [11]. Although these instruments are well validated and part of standard of practice, they offer only a “snapshot” of performance during clinical visits.

Wearable inertial measurement units (IMUs) have emerged as a promising technology for augmenting clinical assessment by enabling objective, high-resolution kinematic measurement of gait in the laboratory, clinic, and community settings [12,13]. Prior research has demonstrated the feasibility of using IMUs to estimate spatiotemporal gait parameters, identify asymmetries, and classify deviation patterns in individuals with lower-limb deficits or altered biomechanics [14,15,16,17,18,19,20,21,22,23,24,25,26,27]. Established composite gait measures, including the Gillette Gait Index, Gait Deviation Index, and Gait Profile Score, summarize multidimensional deviations from normative three-dimensional kinematic data [28,29,30]. These laboratory-based measures serve important purposes, but generally require instrumented motion analysis and normative reference data. The present work addresses a different use case: generating a portable, step-wise summary score from clinically interpretable IMU features using classifiers trained against therapist-observed gait deviations.

To address this gap, we developed a machine learning framework that integrates step-wise IMU features, calculated at heel strike from left–right step pairs, into a continuous Gait Goodness Score (GGS), accompanied by a classification of the predominant gait deviation type. The GGS combines information from multiple temporal, spatial, symmetry-related, and joint-motion features into a single normalized score, representing the relative evidence for non-deviated gait versus the most prominent targeted deviation. It was intended to complement, rather than replace, individual spatiotemporal parameters and established clinical outcome measures. Initial development and internal validation were performed using data from a supervised clinic in association with the Amputee Coalition, where subjects completed supervised walking tasks under controlled conditions.

The objective of the present study is to extend and rigorously evaluate the GGS metric through prospective, supervised clinical validation. Specifically, we examined the performance of the GGS and deviation classification outputs during standardized walking tests (10mWT) conducted in clinic under the supervision of a physical therapist and alongside established functional outcome measures. By anchoring validation to clinically supervised testing and widely accepted reference instruments, this study assessed the construct validity and clinical relevance of the GGS metric while minimizing confounding variables associated with home-based data collection. Importantly, while the broader system architecture supports longitudinal monitoring and real-time feedback, those components are beyond the scope of this paper. This work focuses exclusively on the development, training, and validation of the GGS and deviation classification metrics, establishing the necessary foundation for future deployment in unsupervised settings.

## 2. Classifier Development: Methods and Results

### 2.1. Gait Goodness and Classifier Development: Training and Methods

#### 2.1.1. Data Collection

A convenience sample of 71 community-ambulating people with a unilateral LLA and 7 people without LLA were enrolled in this study. Subjects with LLA were recruited from the Amputation Coalition National Conference in Greensboro, NC. The seven participants without LLA were recruited as a convenience sample of adults from the Functional Outcome Research and Evaluation (FORE) Center at the University of Miami Department of Physical Therapy. They provided development examples of non-deviated gait but were not intended to establish a population-level normative reference. All subjects provided informed consent to participate in this study, which was approved by the Miami Veterans Affairs Healthcare System institutional review board. Inclusion criteria included: age of 18 to 80 years old, unilateral LLA at the level of the shank, knee, or thigh, and a well-fitting prosthetic socket. Anthropometric measurements, including height, weight, waist circumference, and lower-limb segment length, were recorded for all subjects.

#### 2.1.2. Instrumentation and Protocol

Subjects were instrumented with knee sleeves containing two IMUs affixed superior and inferior to the anatomical and/or prosthetic knee joint. Motion kinematics and video were simultaneously collected and transmitted to a custom iPad application (App) during two 10 m walk (10mWT) trials at self-selected walking speed. The 10mWT was performed on a level surface, with tape lines used to demarcate the 2 m and 8 m points. As the participant crossed these points, the physical therapist (PT) used the iPad App to manually “identify” the events for subsequent processing. Only data collected between the 2 m and 8 m marks were included in the final analyses to exclude steps within the acceleration and deceleration zones.

**Figure 1:**
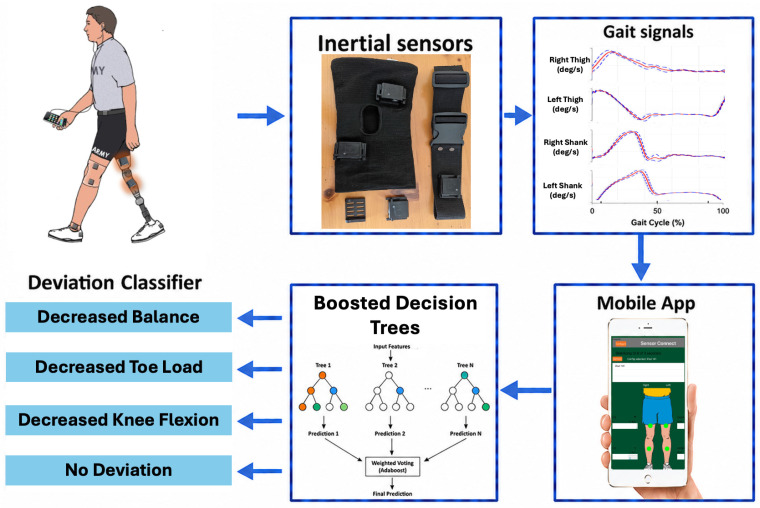
Inertial sensors collected kinematic information, processed on an iOS app. A collection of AdaBoost decision tree ensembles identified the most prominent gait deviation according to the spatiotemporal and joint-motion gait parameters exhibited by the participant.

This dataset was used exclusively for GGS metric development and training and was not used for prospective validation. The objective of this phase was to establish a robust mapping between step-wise gait features and clinically meaningful indicators of gait quality and deviation patterns. The data collection process is shown in Figure 1.

#### 2.1.3. IMU Sensor System and Online Calibration

Gait data were collected using a four-sensor wireless IMU system that was placed bilaterally by either donning a sensory embedded knee sleeve or mounting the sensors directly on the prosthesis using Velcro adhesive and elastic straps. Each IMU recorded tri-axial linear acceleration and angular velocity. Data were transmitted to an iPad at a sampling rate of 50 Hz via Bluetooth Low Energy (BLE). Sensor data were interpolated, filtered, and up-sampled on-device before storage. All raw and processed data were uploaded to a HIPAA- and FIPS 140-2–compliant cloud infrastructure using PHP web services and a PostgreSQL database.

IMUs were calibrated prior to use, and their placement was determined based on the need to minimize motion artifacts arising from soft tissue displacements during ambulation [14]. Anatomical locations that were both bony and flat were selected. In software, we rotated the IMUs’ frames of reference to align with the participant’s body such that the *y*-axis aligned with gravity (Superior–Inferior (SI)), the *x*-axis with the Medial–Lateral (ML) axis, and the *z*-axis with the Anterior–Posterior (AP) axis.

#### 2.1.4. Gait Parameters and Target Deviations

We deliberately used clinically validated step-level temporal, spatial, symmetry-related, and joint-motion parameters rather than directly modeling the complete raw IMU time series. This feature-based representation was selected to preserve clinical interpretability, permit traceability of classifier outputs to recognizable gait characteristics, and support low-latency execution on the mobile platform. Gait phase detection was performed using gyroscopic data and used to calculate the following: single-limb support (SLS) times, double-limb support (DLS) times, step lengths, stride lengths, gait velocities (GVs), gait cycle times (GCTs), and peak knee angle during flexion. These algorithms were previously validated against Vicon and Tekscan Matscan systems as criteria measures [14,15,16]. The classifier targeted three amputee gait deviations:

1. Decreased Balance over the prosthesis (DecBal-p): Reduced SLS time on the prosthetic limb relative to the sound limb.
2. Decreased Toe load (DecToe-p): Early initiation of swing on the prosthetic side.
3. Decreased Knee flexion (DecFlex-p): Reduced peak swing-phase knee flexion.
4. No Deviation (NoDev): No identifiable deviation was present.

#### 2.1.5. Ground Truth Gait Classification

Walking videos were independently reviewed by five licensed PTs. Each rater identified the presence or absence of the three target deviations for each subject walk. Each walk could be scored as follows: DecBal-p, DecFlex-p, DecToe-p, a combination of two or three of these deviations, or NoDev. Majority consensus was used to determine the final gait deviation labels for classifier development. Walking velocity, AMP score, TUG time, K-level, and the other outcomes subsequently examined in the prospective cohort were not used as classifier targets.

#### 2.1.6. Feature Extraction and Data Pre-Processing

IMU data were processed in real time to compute gait parameters at each detected heel-strike event. Features were pooled into stride level tuples, combining parameters from the current step and the preceding contralateral step. Each feature vector represented a complete gait cycle, including sound and prosthetic limb metrics. A total of 2778 stride pairs were included in the training and validation dataset (averaging approximately 36 per subject). Table 1 details the extracted features.

#### 2.1.7. Classifier Architecture and Implementation

The machine learning framework consisted of four binary AdaBoost decision tree ensembles, one for each gait category: DecBal-p, DecToe-p, DecFlex-p, and NoDev. AdaBoost with shallow trees was selected to provide nonlinear classification while retaining modest computational requirements, reproducible on-device execution, and a direct connection between decisions and clinically interpretable input features. Limiting each weak learner to a shallow tree also constrained model complexity, which helped to limit overfitting [31].

**Figure 2:**
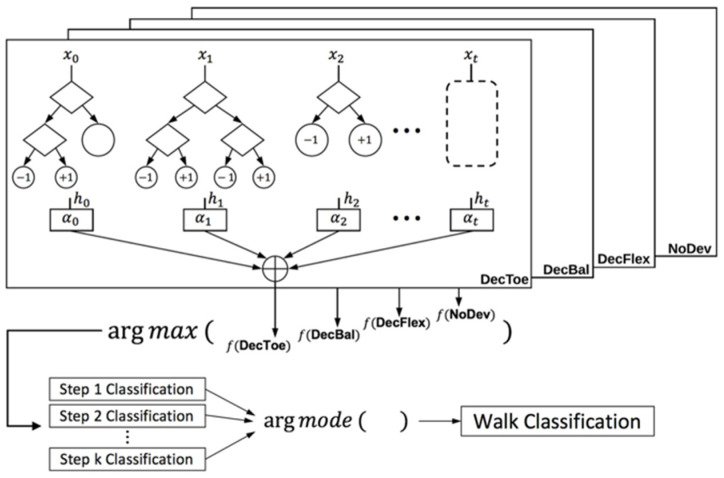
Classifier decision process. The subset of features (*x_t_*) for each individual tree, *t*, generates a decision (*h_t_*) that is weighted (*α_t_*) and summed to produce a margin (*f_dev_*) for that class. The maximum margin is selected as the prominent deviation for that step. Bagging of steps produces an aggregate walk classification.

For *M* training samples, the training process underwent 300 iterations, one per weak learner, *t*. A distribution of weights, *α_t_*(*m*), was initialized to *α_1_*(*m*) = 1/*M* for the *m* = 1, …, *M* training samples. In each iteration, a tree was trained on the weighted samples to generate a weak hypothesis, *h_t_*, that minimized the weighted classification error, *ε_t_*, under the criterion *ε_t_* < 0.5 to ensure performance exceeded chance. The resulting ensemble classification *H*(***x***), as in (1), is defined as:

(1) $$H\left(x\right)=sign\left(f_{dev}\left(x\right)\right),\;$$

where $\alpha_{t}=\;\frac{1}{2}ln(\frac{1-\varepsilon_{t}}{\varepsilon_{t}})$ is the weight assigned to each weak learner and $h_{t}\left(x\right)\in [-1,\;1]$ is the classification of each weak learner. The margin, *f_dev_*(***x***), is defined, as in (2), for each classifier as:

(2) $$f_{dev}\left(x\right)=\sum_{t=1}^{T}\alpha_{t}h_{t}\left(x\right),\;$$

where the margin is a function of the feature set, ***x***, depending on the decision, *h_t_*(***x***), and normalized weight, *α_t_*, of each tree, *t*, within the total set of trees, *T*. Each ensemble used *T* = 300 weak tree learners with no more than five split nodes. The overall ensemble architecture is shown in Figure 2.

The classifier was implemented directly within the iPad app using Apple GameKit, enabling parallel tree inference on the device GPU. The model was executed in 33 ± 8 ms, supporting real-time gait classification during ambulation. Models were centrally stored and distributed via secure cloud infrastructures.

#### 2.1.8. Classifier Training and Testing

Classifier performance was assessed using participant-level hold-out validation. A predefined set of 14 participants representing target and non-target gait patterns was withheld for validation. Each participant was evaluated in a separate holdout iteration. For each held-out participant, all strides from that participant were excluded from model training and used only for testing; no participant contributed stride-level data to both the training and test sets. Both in-class and out-of-class examples were included with balanced representation, and performance was evaluated using accuracy and F1-score. Sensitivity was computed from in-class classifications, and specificity from out-of-class classifications. Lastly, to evaluate robustness, white Gaussian noise was added to each gait feature at levels ranging from 0.25× to 2× the observed standard deviation for each feature. Classification accuracy degradation was then quantified relative to baseline performance. These analyses evaluated internal performance during algorithm development. They were distinct from the subsequent prospective clinical validation, in which the completed classifier models were fixed and applied without retraining or optimization to an independent multisite cohort.

### 2.2. Gait Goodness and Classifier Development: Results

#### 2.2.1. Subject Demographic Information

Seven non-amputee individuals were recruited with a mean age of 31 (±9) years, height of 168 (±7) cm, and body mass of 69 (±6) kg. Seventy-one individuals with LLA were recruited and subjects had a mean age of 49 (±14) years, height of 175 (±10) cm, and body mass of 78 (±21) kg, with a mean amputation age of 11 (±10) years. The LLA cohort had a mean self-perceived prosthetic mobility (Prosthetic Limb Users Survey of Mobility or PLUS-M) score of 52 (±7), indicating they performed at the 73rd percentile of prosthetic-ambulating individuals with LLA. A certified prosthetist classified the functional level of each subject using the Medicare Functional Classification Level (K-Level). In summary, the cohort consisted of the following: 13 K2-level, 53 K3-level, and 5 K4-level ambulators. Regarding amputation level, 38 had transtibial LLA, 2 had knee disarticulation LLA, and 31 had transfemoral LLA. According to the panel of 5 PT assessors, the LLA cohort walked with the following gait deviations: NoDev (*n* = 13), Other/Unknown (*n* = 15), DecToe-p (*n* = 15), DecFlex-p (*n* = 0), DecBal-p (*n* = 2), DecToe-p + DecFlex-p (*n* = 3), DecBal-p + DecToe-p (*n* = 16), DecToe-p + DecFlex-p + DecBal-p (*n* = 14).

#### 2.2.2. Feature Set Reliability

Table 2 presents the temporal and spatial gait metrics calculated during trials 1 and 2 of the 10mWT. Across subjects with LLA, expected asymmetries were observed, including a longer step length and shorter stance time with the prosthetic limb and a shorter step length and longer stance time with the sound limb. Participants’ velocity and gait cycle times were within the normal range of non-amputee ambulators. The estimated peak knee angle for the prosthetic limb was within the range of non-amputee ambulators but was less than that of the sound limb.

Importantly, Intraclass Correlation Coefficient (ICC) values for all temporal and spatial parameters demonstrated excellent reliability between trials for the sound and prosthetic sides. This supports the stability of the extracted feature set and its suitability for machine learning classifications. These findings establish that the classifier inputs were not only clinically meaningful but also highly repeatable under standardized walking conditions.

#### 2.2.3. Classifier Performance and Cross-Validation Results

Within the development dataset, step-level F1 scores ranged from 92% to 100% across DecToe-p, DecBal-p, DecFlex-p, and NoDev classifiers, as shown in Table 3. These results indicate strong agreement with clinician consensus labels for participants withheld from model training. They represent subject-level internal development performance and should not be interpreted as independent external validation of the classifiers.

To assess development-phase walk-level performance, step-wise classifications were aggregated by majority vote across all analyzed steps within a trial. Within the subject-level cross-validation framework, all aggregated walk classifications were concordant with the clinician consensus label. This result reflects the evaluated development sample and aggregation procedure, and while individual step misclassifications may occur, the dominant gait pattern within a walk was consistently and correctly identified.

#### 2.2.4. Sensitivity of Classifier Performance to Noise

**Figure 3:**
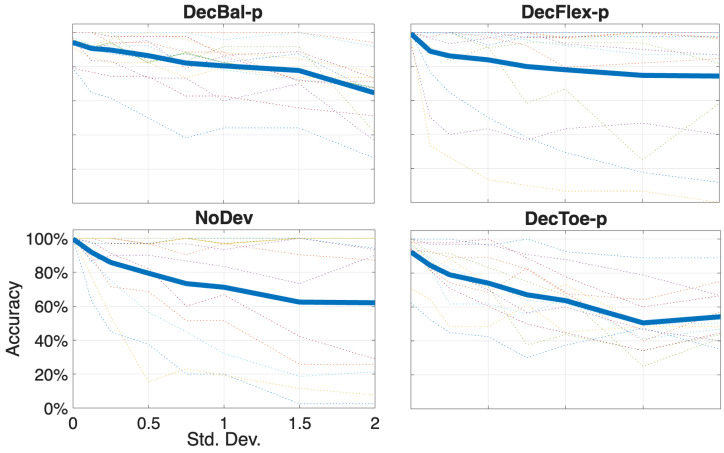
Noise injection stress test within the feature set to examine robustness. Thin dashed lines are individual subject-level holdout results, and dark thick curves represent the mean across holdouts at each noise injection level. Injected white Gaussian noise ranged from 0.25 to 2.0 times the observed standard deviation of each feature.

Classifier accuracy exhibited a gradual degradation as the magnitude of additive white Gaussian noise increased, as shown in Figure 3. At a noise level equal to one standard deviation of the observed feature distribution, the mean in-class accuracy decreased by approximately 27%. At two standard deviations, accuracy decreased by approximately 36%. Despite this decline under extreme conditions, classifier outputs remained stable at low-to-moderate noise levels. These findings indicate limited degradation at lower simulated noise levels and progressively greater degradation at higher levels. Because artificial feature-level noise does not reproduce all sources of sensor placement error or community walking variability, this analysis should be interpreted as a controlled stress test rather than a complete external assessment of robustness.

### 2.3. Summary of Development-Phase Results

Collectively, the development-phase results demonstrate that the IMU-based gait deviation classifiers achieved high internal subject-level agreement with clinician-observed gait deviations while operating with sufficient computational efficiency for mobile deployment. These findings established the classifier framework used to construct the GGS but did not, by themselves, establish external clinical generalizability. Clinical validity was, therefore, evaluated separately in the prospective multisite cohort using fixed models and independent clinical outcomes.

Notably, beyond binary classification performance, the boosted ensemble architecture yields continuous decision margins for each gait class, reflecting the relative confidence of the classifier’s predictions. These margins are consistently stable within individual trials, discriminate effectively between normal and deviated gait patterns, and degrade predictably under noise perturbation. As such, they provide a principled, quantitative basis for representing gait quality along a continuum rather than as a categorical outcome alone, motivating their use in the construction of a continuous gait quality score.

## 3. Prospective Validation: Methods and Results

### 3.1. Validation Methods

#### 3.1.1. Study Design, Subjects, and Sites

This study employed a prospective, observational validation design conducted in supervised clinical environments. Data were collected across multiple outpatient rehabilitation settings within the U.S. Department of Defense (DoD) and Veterans Affairs (VA) Health System. Institutional review board (IRB) approval was granted by the VA Central IRB, Walter Reed National Military Medical Center (WRNMMC), and participating VA facilities (Bruce W. Carter VA Medical Center, Miami, FL; Washington DC VA Medical Center, Washington, DC; Boston VA Medical Center, Boston, Massachusetts; Richmond VA Medical Center, Richmond, VA; VA Indiana Healthcare System, Indianapolis, IN; VA Eastern Colorado Healthcare System, Aurora, CO). All subjects provided written informed consent prior to enrollment. Clinical testing and data collection were performed by licensed physical therapists in accordance with approved study protocols. List of sites used in conduction of this study can be found in Appendix A.

Subjects consisted of medically stable adults (aged 18 to 80 years) with unilateral or bilateral LLA, including transtibial, transfemoral, knee disarticulation, and Symes amputation. Inclusion required subjects to be at least one-month post-initial prosthetic fitting and cleared for home-based prosthetic use. At the time of testing, all subjects wore a well-fitting, properly aligned prosthesis. Exclusion criteria included neurological conditions affecting gait, cognitive impairment limiting the ability to follow testing instructions or use study technology, medical instability, or other comorbid conditions precluding safe ambulation. Subjects were primarily recruited from amputee clinics and outpatient physical therapy departments. All walking tasks were performed under direct supervision of a licensed physical therapist. The prospective validation cohort was restricted to individuals with LLA and did not include a concurrent nondisabled comparison group, because its primary purpose was to evaluate clinical relationships within the intended rehabilitation population rather than to establish normative GGS values.

#### 3.1.2. Equipment, Apparatus, and Outcomes

Gait data were captured using the previously described ReLOAD mobile application and its integrated wearable IMU sensor system [32]. Sensors were mounted on the lower limbs according to standardized placement and alignment procedures previously validated for spatiotemporal gait analysis in individuals with LLA [14,15,16]. To preserve independence between development and prospective clinical validation, all classifier models and algorithm parameters were fixed before analysis of the prospective cohort and were not retrained, modified, or recalibrated during this study.

The primary performance-based outcome used for validation was the 10mWT, administered at both self-selected (comfortable) and fast walking speeds following standardized clinical protocols. Additional clinical measures collected during the same visit included AMP score, K-level, demographics, and amputation-related characteristics. During each walking trial, the classifier generated step-wise outputs, including deviation-specific ensemble margins and the derived GGS. These outputs were aggregated across steps to produce condition-level summaries for subsequent statistical analyses.

#### 3.1.3. Derivation of Gait Goodness Score

The machine learning framework produces four normalized decision scores for each analyzed stride: one corresponding to non-deviated gait (NoDev) and three corresponding to specific targeted gait deviations (DecToe-p, DecBal-p, and DecFlex-p). Each score is derived from a separate AdaBoost ensemble and is bounded within the interval [0, 1], with larger values indicating stronger support from that classifier. These outputs are classifier decision scores and are not interpreted as calibrated posterior probabilities.

Although the individual classifier outputs support categorical gait deviation identification, a scalar measure is required to summarize their relative pattern along a continuum suitable for prospective clinical validation. To this end, we define a Gait Goodness Score (GGS) as the contrast between the NoDev classifier output and the largest output among the three deviation classifiers. For each step pair, the raw GGS is defined as the difference between the normalized NoDev decision score and the largest normalized decision score among the three targeted deviation classifiers:

(3) $$GGS_{raw}=f_{NoDev}-\max\left(f_{DecToe},f_{DecBal},f_{DecFlex}\right),$$

where *f* denotes the margin of each classifier output. This formulation yields a higher raw GGS when the NoDev classifier provides stronger relative support than any of the deviation classifiers and a lower raw GGS when one of the deviation classifiers provides stronger relative support.

Unlike binary classification labels, the GGS retains continuous variation in the relative classifier outputs. Changes in the underlying spatiotemporal and joint-motion features can, therefore, alter the score, even when the predominant categorical classification remains unchanged. Because the raw GGS is bounded within [−1, +1], it is linearly normalized to the interval [0, 1] for interpretability and consistency with clinical scoring conventions:

(4) $$GGS=\frac{GGS_{raw}+1}{2}.\;$$

Under this normalization, a value closer to 1 indicates strong confidence in non-deviated gait, while a value approaching 0 indicates strong confidence in at least one gait deviation. A value of 0.5 represents the “break-even” point, at which support for non-deviated gait equals support for the most dominant deviation.

The GGS does not require the classifier outputs to be interpreted as calibrated probabilities. It does, however, compare their relative magnitudes. To support this use, all four ensembles were constructed from the same development cohort and feature space and used the same boosting procedure, ensemble size, weak-learner depth, and output normalization. These design constraints place the outputs on a common bounded numerical scale, although they do not establish probabilistic calibration or identical score distributions. The clinical interpretation of the resulting composite score was, therefore, evaluated empirically in the independent prospective cohort through its relationships with established clinical measures and functional groups.

### 3.2. Validation Results

#### 3.2.1. Participant Demographics

A total of 120 subjects with unilateral or bilateral LLA were included in the prospective validation protocol. Subjects represented a broad range of functional mobility levels, amputation levels, amputation types, and ages typical of a clinical prosthetic rehabilitation population. Among this cohort, 69 completed the Amputee Mobility Predictor (AMP), enabling the assignment of functional K-levels. A total of 93 subjects performed a standardized 10mWT under physical therapist supervision. Of these, 83 were instrumented with the IMU sensor system, and, therefore, contributed valid step-level data for computation of the GGS. Finally, 45 subjects completed the instrumented 10mWT and AMP testing, and 42 completed the instrumented 10mWT and TUG testing.

During administration of the instrumented 10mWT, to eliminate acceleration and deceleration phases, a GGS was computed for each step, excluding the first three and last three steps of each trial. All remaining steps were aggregated for analysis. Subjects were stratified by K-level, age, and amputation level for group-based analyses. While additional fast-walking speed trials were collected, they were excluded from primary analysis to maintain focus on gait during representative, comfortable ambulation.

#### 3.2.2. Distribution of GGS and Outcome Values—Descriptive Statistics

During self-selected walking, the GGS demonstrated a mean value of 0.49 ± 0.05, with observed values spanning a range of [0.37, 0.59]. The distribution of GGS values was continuous and exhibited sufficient variability to support both group-based and correlational analyses, as shown in Table 4.

#### 3.2.3. GGS by K-Level

The GGS values differed significantly with a Tukey–Kramer correction among K-levels during self-selected walking (F = 5.74; *p* < 0.01), with the exclusion of K1 due to insufficient sample size. Subjects with higher functional classification exhibited progressively higher GGS values, reflecting a positive correlation between the score and improved overall gait quality, as shown in Table 5. Post hoc comparisons revealed statistically significant differences between all K-level groups with the exception of K3 and K4, with effect sizes ranging from moderate to large.

#### 3.2.4. GGS by Amputation Level, Age, and Gender

No significant difference in GGS between genders (68 male; 15 female) was found. However, a significant difference in GGS was observed across amputation level. Subjects with transtibial amputation (*N* = 45) demonstrated significantly (*p* = 0.0012) higher GGS values (0.50 ± 0.04) than those with transfemoral amputation (*N* = 34) during self-selected walking (0.47 ± 0.05). Age also demonstrated a statistically significant association with GGS, where advancing age was associated with modest reductions in GGS (*N* = 83; *ρ* = −0.259; *p* < 0.05). However, substantial variability was observed across the age ranges, and when examining age groups, as shown in Table 6, with a Tukey–Kramer correction applied, significant differences were not observed.

#### 3.2.5. Associations Between GGS and Clinical Performance Measures

The GGS was associated with objective walking performance: higher GGS values were correlated with faster self-selected walking velocity (*N* = 83; *ρ* = 0.675; *p* < 0.0001) and were inversely correlated with the time required to complete the 10mWT (*N* = 83; *ρ* = −0.537; *p* < 0.0001). These findings demonstrate an expected relationship between classifier-derived score and overall walking performance; they do not establish that the GGS is independent of walking velocity.

A large positive correlation was also observed between GGS and the AMP score (*N* = 45; *ρ* = 0.674; *p* < 0.0001), while an inverse moderate-to-strong correlation was observed with TUG test time (*N* = 42; *ρ* = −0.522; *p* < 0.001). A modest positive correlation was observed with the PLUS-M instrument (*N* = 61; *ρ* = 0.275; *p* < 0.05). While a statistically significant association was not observed between GGS and the ABC scale, a trend nearing significance was observed (*N* = 32; *ρ* = −0.310; *p* = 0.086). It was noted that the relationship magnitude was larger than with the PLUS-M, with a drop in sample size resulting in low power.

### 3.3. Summary of Validation-Phase Results

Collectively, these results demonstrate that the GGS differs across clinically relevant functional classifications and amputation level and is associated with walking performance during supervised self-selected walking. The cross-sectional design does not establish the magnitude of within-person change that should be considered clinically important. The GGS exhibited strong associations with established performance-based clinical measures, supporting its validity as a quantitative summary metric of gait quality in individuals with lower-limb amputation.

## 4. Discussion

This study sought to prospectively evaluate an IMU-based gait deviation classifier and a derived GGS during supervised clinical walking in individuals with LLA. We report on three primary findings. First, the classifiers demonstrated high agreement with therapist-observed gait deviation labels during subject-level internal development testing. Second, when the completed models were fixed and applied without retraining in an independent prospective cohort, the GGS differed across functional mobility levels (i.e., K-level groups) and was significantly associated with established performance-based measures, including walking speed, AMP score, and TUG time. These prospective findings provide the principal evidence of clinical validity presented in this study and should be distinguished from the development-phase classification performance estimates. Third, not all clinical measures demonstrated significant associations with the GGS; specifically, the ABC score showed no correlation, and the PLUS-M was only fairly correlated. This suggests that objective gait quality, functional, and prosthetic mobility are strongly related, yet objective gait quality may not always align with a patient’s perceived confidence in their balance and/or mobility.

The study provides two complementary forms of validation. During development, each classifier was evaluated against consensus observational gait analysis labels assigned by licensed physical therapists; thus, the construct used to build the GGS was the presence or absence of clinically observed gait deviations rather than walking speed or functional outcome scores. During prospective validation, the fixed GGS was evaluated against independently collected clinical measures and functional classifications in a separate multisite cohort. This two-stage structure supports the interpretation that the score reflects clinically observed gait deviation patterns that also relate to overall mobility. The GGS is not intended to represent a single intrinsic physiological variable; rather, it is a composite clinical score derived from validated biomechanical measurements and classifiers trained against therapist-observed gait deviations, with its clinical meaning established through prospective validation against independent functional outcomes.

Objective gait assessment in individuals with LLA has traditionally relied on laboratory-based motion capture, instrumented walkways, and expert observational analysis. While these methods provide detailed biomechanical data, they are not scalable to community or home-based use. Wearable IMUs have demonstrated validity and reliability for estimating spatiotemporal gait parameters; however, most prior work has focused on discrete parameters rather than synthesizing multi-parameter information into a single, interpretable metric.

The present study extends this literature by training classifiers against therapist-observed gait deviations using clinically interpretable spatiotemporal and joint-motion parameters and then combining their relative outputs into a continuous scalar GGS. Unlike an individual parameter such as walking velocity, step length, or stance time, the GGS summarizes evidence across multiple gait characteristics while preserving the deviation-specific classifier outputs. The observed associations between GGS, K-level, AMP, and walking speed align with the hypothesis that higher functional mobility corresponds to more symmetric and efficient gait patterns that are absent of gait deviation.

A key methodological feature of this framework is the controlled and consistent structure of the four classifier ensembles. Each used identical architectures, feature sets, number and depth of weak learners, boosting procedures, and output normalization. This consistency permitted the outputs to be compared as bounded decision scores. It does not, however, imply that the outputs are calibrated probabilities or that their empirical distributions are identical. Accordingly, the score’s clinical interpretation rests on its prospective performance in an independent cohort.

Contemporary sequence-based approaches, including convolutional, recurrent, and transformer architectures may capture temporal dynamics that are not represented fully by engineered features. The purpose of the present study, however, was not to establish superiority over all available machine learning architectures; rather, the model was designed for interpretable, low-latency execution within an existing mobile rehabilitation platform. This design involves a tradeoff between richer raw signal representation and clinical interpretability, computational efficiency, and deployability.

The use of classifier margins combined into a continuous metric offers several practical advantages. First, they preserve information about the relative support for each gait deviation class across strides. Second, aggregation of step-wise GGSs across walking trials reduces sensitivity to single-step variability while preserving deviation-specific information. Furthermore, in the prospective validation cohort, the GGS demonstrated construct validity through its associations with established performance-based measures. This suggests that ensemble margins are sufficiently stable and comparable across classifiers to serve as the foundation for a composite score. From a rehabilitation perspective, such a score may enable objective monitoring of gait quality alongside standard outcome measures, potentially improving clinical decision-making, stratification, and monitoring.

### 4.1. Limitations

Several limitations of the present study should be acknowledged. First, validation was conducted exclusively during supervised clinical testing. Although this controlled environment strengthens internal validity, these results may not fully generalize to unsupervised, home-based or community-based walking contexts where environmental factors are less predictable.

Second, the AdaBoost framework was not benchmarked against deep learning or other raw time-series models. The present results, therefore, support the clinical validity and deployability of the selected framework without establishing it as the optimal classifier architecture for all gait-assessment applications.

Third, participant recruitment was not balanced against a healthy control group, and the self-selection process may have introduced selection bias; the gait characteristics of individuals who declined participation could differ from those who enrolled, potentially impacting the generalizability of the findings. The development phase included only seven participants without LLA, and the prospective validation phase lacked a concurrent nondisabled reference group. Therefore, although nondisabled examples contributed to development of the NoDev classifier, this study cannot define a population-level normative or “ideal” GGS value. Future studies should include a larger and more diverse nondisabled reference cohort.

Fourth, the current analysis focused on cross-sectional group comparisons and associations. As such, the longitudinal responsiveness of the GGS and its ability to track clinical changes over time were not evaluated in this phase. Additionally, this study was not designed to establish test–retest measurement error, responsiveness, minimal detectable change, or a minimal clinically important difference for the GGS. Consequently, the present results support interpretation of between-participant and between-group differences but do not yet provide a threshold for clinically significant longitudinal change.

Fifth, the present analysis did not stratify participants by prosthetic foot or knee technology. Accordingly, the study cannot determine whether the GGS or individual deviation-classifier outputs differ systematically among hydraulic, microprocessor-controlled, energy-storing-and-return, or other prosthetic component categories.

Sixth, several of the classifier inputs were influenced by walking speed. Although the classifiers were trained against therapist-observed gait deviations rather than velocity, the present cross-sectional analyses cannot fully disentangle the effects of walking speed from other gait characteristics. Accordingly, the GGS should be interpreted as a composite measure of overall gait quality rather than a speed-independent measure of gait.

Finally, while multiple clinical measures were collected, the lack of significant associations with certain subjective instruments highlights the multidimensional nature of mobility. This suggests that while the GGS is a robust indicator of biomechanical gait quality, it captures specific performance-based aspects that are distinct from a patient’s perceived confidence or self-reported functional status.

### 4.2. Future Directions

Although the present study focused on supervised clinical validation during standardized walking, the system architecture was intentionally designed to extend beyond the clinic. The classifier operates in real time and generates step-wise margins within milliseconds to produce a continuous GGS that can be aggregated over multiple steps. This computational efficiency and portability enable deployment within home or community environments without the need for modification of the trained model.

The GGS offers significant advantages for remote monitoring. Rather than relying on periodic clinical visits and standard outcome measures, clinicians could track gait quality longitudinally during unsupervised ambulation. Changes in GGSs may reflect improvement, plateaus, or regressions in mobility, potentially identifying issues related to prosthetic fit, alignment, or secondary health effects. Critically, the system may detect these subtle shifts in gait quality before functional decline becomes clinically apparent.

In addition, real-time access to deviation-specific margins creates a foundation for closed-loop feedback interventions. When deployed in a mobile application, the classifier outputs can be mapped to auditory or haptic cues that reinforce non-deviated gait or prompt correction of the most prominent deviation. This framework supports adaptive, personalized rehabilitation that extends therapy outside the clinical setting. Future longitudinal studies should establish GGS test–retest reliability, standard error of measurement, and responsiveness to rehabilitation. Subsequent studies should then evaluate whether GGS-guided monitoring and feedback decrease gait deviations and improve long-term functional outcomes.

## Figures and Tables

**Table 1:** Description of temporal and spatial features collected each stride.

Feature	Description
Step velocity	Linear velocity of the limb during swing phase
Step length	Distance from the toe of one foot to the heel of the contralateral foot
Single-limb stance (SLS) time	Duration of the stance phase with a single foot planted on the ground
Double-limb stance (DLS) time	Duration of the stance phase with two feet planted on the ground
Gait cycle time (GCT)	Duration from heel strike to heel strike of the ipsilateral foot, averaged over both limbs
Body’s velocity	Linear velocity of the center of mass
Peak knee flexion	Maximum flexion of knee angle during swing phase

**Table 2:** Descriptive statistics and repeatability of temporal and spatial gait features.

Metric	Limb	Trial 1		Trial 2		ICC
		Mean	SD	Mean	SD	
Step Velocity (cm/s)	Prosthetic	117	31	119	32	0.99
	Sound	116	31	119	32	0.97
Step Length (cm)	Prosthetic	70	16	70	17	0.96
	Sound	60	13	61	13	0.96
SLS Time (s)	Prosthetic	0.35	0.035	0.35	0.035	0.85
	Sound	0.41	0.038	0.40	0.039	0.86
DLS Time (s) ^1^	Prosthetic	0.18	0.057	0.18	0.060	0.92
	Sound	0.19	0.062	0.19	0.061	0.91
GCT (s)	Prosthetic	1.13	0.13	1.13	0.13	0.97
	Sound	1.13	0.13	1.12	0.13	0.99
BV (m/s)	Prosthetic	1.17	0.30	1.18	0.32	0.98
	Sound	1.16	0.30	1.18	0.31	0.97
Peak Knee Flexion (deg)	Prosthetic	60	11	60	12.0	0.97
	Sound	72	16	72	16.1	0.98

^1^ Measured limb is the trailing limb.

**Table 3:** Cross-validation performance by gait deviation classifier.

Classifier	Sensitivity (%)	Specificity (%)	F1
DecBal-p	91	98	0.94
DecToe-p	93	92	0.92
DecFlex-p	100	98	0.99
NoDev	100	100	1.00

**Table 4:** Participant demographics and self-selected walking trial characteristics.

Variable	Mean (±Std. Dev.)	Median	[Min., Max.]
Age (years)	52.6 (15.5)	57	[19, 80]
Time since amp. (years)	9.3 (13.7)	3.2	[0.3, 56.3]
Velocity (m/s)	1.03 (0.30)	1.10	[0.20, 1.60]
Time (s)	6.6 (3.2)	5.5	[3.7, 27.3]
GGS	0.49 (0.05)	0.50	[0.37, 0.59]
AMP	38.1 (6.9)	39	[16, 47]

**Table 5:** Characteristics of GGS, stratified by K-level.

K-Level (AMP)	*N*	Mean	Std. Dev.
K1	5	0.44	0.06
K2	20	0.45	0.04
K3	23	0.51	0.04
K4	21	0.52	0.03

**Table 6:** Characteristics of GGS, stratified by age.

Age	*N*	Mean	Std. Dev.
16–39	11	0.53	0.03
40–55	20	0.51	0.05
55–64	19	0.47	0.05
65+	19	0.47	0.03

## Data Availability

The data presented in this study are not public due to privacy reasons.

## Abbreviations

The following abbreviations are used in this manuscript:

GGS	Gait Goodness Score
LLA	Lower-limb amputation
TUG	Timed up-and-go
10mWT	Ten-meter walk test
AMP	Amputee mobility predictor
PLUS-M	Prosthetic limb users survey of mobility
NoDev	No deviation
DecBal-p	Decreased balance over the prosthesis
DecToe-p	Decreased prosthetic toe load
DecFlex-p	Decreased prosthetic peak swing flexion
SLS	Single limb stance
DLS	Double limb stance
GCT	Gait cycle time

## Appendix A

The following sites were used in the conduction of this study:

WRNMMC	Walter Reed National Military Medical Center
MVAHS	Miami VA Healthcare System
RVAMC	Richmond VA Medical Center
WVAMC	Washington VA Medical Center
RMRVAMC	Rock Mountain Regional VA Medical Center
VAIHS	VA Indiana Healthcare System
VABHS	VA Boston Healthcare System
